# In Silico Perturbome Analysis Reveals Conserved Genes and Drug–Target Interactions in *Pseudomonas aeruginosa*, *Escherichia coli*, and *Staphylococcus aureus* in the Response to Stress

**DOI:** 10.3390/pathogens15070665

**Published:** 2026-06-25

**Authors:** Jose Arturo Molina-Mora, Ravi Kant

**Affiliations:** 1 Centro de Investigación en Enfermedades Tropicales, Centro de Investigación en Hematología y Trastornos Afines & Facultad de Microbiología, Universidad de Costa Rica, San Jose 11501-2060, Costa Rica; 2 Faculty of Applied Sciences & Biotechnology, Shoolini University, Solan 173229, Himachal Pradesh, India; ravikant2@shooliniuniversity.com

**Keywords:** perturbation, perturbome, stress response, bacteria, orthologs, molecular docking, interactome, *Pseudomonas aeruginosa*, *Escherichia coli*, *Staphylococcus aureus*

## Abstract

Background: Bacterial adaptation to environmental and chemical stress involves coordinated, system-level responses collectively described as perturbome. Understanding conserved elements within core perturbomes may reveal strategic vulnerabilities for antimicrobial development. Methods: In this study, we implemented an integrative framework combining functional and comparative genomics, drug–target interactions and molecular docking to prioritize conserved stress-response targets in *Escherichia coli*, *Pseudomonas aeruginosa*, and *Staphylococcus aureus*. Results: A total of 147 genes from previously defined core perturbomes were analyzed through interactome reconstruction and functional enrichment. Interactome and functional analyses revealed significant connectivity and functional clustering, primarily associated with molecule biosynthesis, translation, transcriptional regulation, and energy metabolism. Orthology-based comparative genomics identified six conserved orthogroups shared across at least two species, representing key stress-adaptive nodes including fatty acid synthesis initiation, metabolic stress buffering, transcription termination (Rho), ATP synthesis, peptidoglycan remodeling, and UDP-glucose-mediated envelope biosynthesis. Drug–target interaction analyses suggested that these conserved proteins are modulated by enzymatic inhibitors, metabolite analogs, or active-site competitors. Structural and docking analyses focused on a selected protein, FabF (β-ketoacyl-ACP synthase II) and confirmed catalytically coherent binding of cerulenin within the active site, with high concordance between experimentally resolved and AlphaFold-predicted structures, supporting the reliability of structure-based prioritization. Conclusions: Overall, the results demonstrate that bacterial stress responses converge on evolutionarily conserved metabolic and regulatory elements essential for homeostasis and tolerance to perturbations, being the first work integrating core perturbome data from different microorganisms. The proposed perturbome-informed framework provides a rational strategy to identify robust, broad-spectrum antimicrobial targets and highlights opportunities for drug repurposing and future experimental validation.

## 1. Introduction

Bacterial adaptation to environmental and chemical stressors is a highly coordinated, multiscale process that underlies both survival and tolerance to perturbations [1]. In this context, the concept of the perturbome has emerged to describe the global molecular and physiological responses triggered by external perturbations such as drug exposure, oxidative stress, or nutrient limitation [2,3]. In bacteria, rather than acting solely on isolated targets, stressor agents typically induce broad metabolic and regulatory reprogramming, highlighting the importance of systems-level analyses for understanding drug action and bacterial resilience [4].

Thus, the core perturbome is defined as a central molecular response to multiple disturbances, functioning as an interconnected network that restores cellular homeostasis and promotes survival under stress [5,6]. From a therapeutic standpoint, dissecting the effect of perturbations provides a powerful avenue to identify vulnerabilities that are not apparent when focusing on single genes or proteins [2,7]. Antibiotics have been shown to provoke widespread changes in respiration, redox balance, and central metabolism, revealing that bacterial survival often depends on network-level buffering mechanisms [1,4,8]. Consequently, integrating functional genomics with systems biology and structural bioinformatics approaches is increasingly recognized as essential for prioritizing robust antimicrobial targets and anticipating adaptive responses [9].

Indeed, recent studies have underscored the importance of understanding the effects of large-scale perturbations across a wide diversity of organisms [10,11,12]. For example, initiatives such as PerturbAtlas aim to provide a standardized metadata platform derived from the reanalysis of public transcriptomic datasets to identify patterns associated with different perturbations, including *Escherichia coli* as one of the 13 models [12]. Also, GPSAdb 2.0 (Atlas of Gene-Perturbation Transcriptomes) works as an open resource for exploring transcriptomic consequences of gene perturbations in human cell lines [10].

In the analysis of perturbomes in prokaryotic models, the architecture of this system has been reported only for three major clinically relevant pathogens. In *Pseudomonas aeruginosa* PAO1, machine learning analyses of transcriptomics datasets identified 46 core response genes associated with multiple perturbations, enriched in biosynthesis, molecular binding, metabolism, DNA damage repair, and aerobic respiration [6]. Similarly, large-scale transcriptomic analyses in *E. coli* and *Staphylococcus aureus* identified compact core perturbomes composed of 55 and 46 genes, respectively, including highly connected hub elements [5]. Functional enrichment and interactome reconstruction linked these signatures to energy and macromolecule metabolism, DNA/RNA and protein turnover, transcriptional regulation, virulence, and signaling pathways, supporting the existence of a conserved multiscale stress-response backbone in bacteria, as defined in other studies [13,14,15,16].

Despite these advances, these studies have been conducted independently. Integrated analytical frameworks are needed to identify shared or conserved determinants across the core perturbomes of different microorganisms and to systematically uncover commonly modulated elements involved in bacterial stress responses [10,11,12]. Orthogroup-based analyses enable the detection of evolutionarily conserved proteins across bacterial taxa, facilitating the prioritization of targets with broad-spectrum therapeutic relevance while minimizing lineage-specific [17], as well as transfer of functional and structural knowledge from model organisms to less characterized pathogens [17,18].

The rapid expansion of structural bioinformatics has further enabled the translation of genomic data into mechanistic hypotheses through protein modeling and molecular docking [4,19]. Drug–target interaction analyses allow the evaluation of binding site accessibility, interaction geometry, and physicochemical feasibility of inhibition, providing a rational filter prior to experimental validation [20]. When interpreted alongside perturbome data, these approaches offer a multilevel view that links molecular binding events to potential system-wide physiological consequences.

A major historical limitation in such analyses has been the lack of experimentally resolved protein structures, particularly for hypothetical proteins that can constitute up to ~40% of bacterial genomes [21,22]. Recent advances in artificial intelligence-based structure prediction, especially AlphaFold, have substantially reduced this gap by generating high-confidence three-dimensional models at the proteome scale [23,24,25]. These developments create new opportunities to explore previously inaccessible regions of the bacterial proteome and to systematically evaluate druggability even in the absence of crystallographic data [26].

Thus, this work aims to investigate drug–target interactions within conserved elements of the core perturbomes of *P. aeruginosa*, *E. coli* and *S. aureus* using functional analysis, orthogroup identification, structural modeling and molecular docking as a framework for rational identification of antimicrobial targets in bacterial organisms.

## 2. Materials and Methods

### 2.1. Data Source and Functional Characterization of Core Perturbome Genes

The core perturbomes of three bacterial models were identified in two previous studies (Supplementary Material S1): 46 genes for *P. aeruginosa* [6], 55 and 46 genes for *E. coli* and *S. aureus*, respectively [5]. As detailed in each previous study, the stressors used to define each perturbome comprise different classes of molecules and chemical compounds with antimicrobial activity or capable of inducing cellular stress. These include antibiotics, phenolic compounds and phytochemicals, bioactive fatty acids, antimicrobial peptides, disinfectants, oxidizing agents, toxic metals, and metabolic inhibitors. Details regarding access to the transcriptomic datasets (GEO series), the specific names of each perturbation, and the corresponding stressor classes are provided in the Supplementary Material S2.

To infer the general relationships between all 147 elements from the three core perturbomes, a characterization of all the genes was performed by ontology annotation and molecular interactions (interactome) using the pipeline reported in [27] with STRINGdb [28], including k-means clustering, while the KEGG functional profile was obtained using KEGG Mapper with the BLAST-KOALA platform [29].

### 2.2. Comparative Genomics to Identify Orthologs and Orthogroups

To identify orthologs and orthogroups between the core perturbomes of the three prokaryotic organisms, annotation files of protein sequences (fasta file) of perturbomes were used to perform the analysis with Orthofinder software v2.5.5 [17] with default parameters to perform all-versus-all Protein BLAST 2.16.0 comparisons (e-value threshold of 0.001), normalizing similarity scores by gene length and phylogenetic distance, identifying reciprocal best normalized hits, and clustering related genes into orthogroups using the MCL algorithm. Functions associated with these orthogenes and orthogroups were retrieved from the Uniprot database [30]. Metrics related to sequence comparison within orthogroups are shared in Supplementary Material S3.

### 2.3. Identification of Drug–Target Interactions for Orthologs and Orthogroups

The Target Sequence tool within the Drugbank database [31] was used to model drug–target interactions, and the subsequent identification of conserved proteins as targets of known drugs. For candidate sequences, specific analyses were performed using the BLAST [32] and Drugbank [31] to gain insights about the biology of interactions and the possible role as modulators of core perturbome elements.

### 2.4. Structural Analysis of Drug–Target Interactions for P0AAI5 (FabF)

After the identification of target proteins, structural analysis was implemented to evaluate the interaction with drugs for a specific candidate. Based on results, the P0AAI5 or FabF (3-oxoacyl-ACP synthase II) gene from *E. coli* was selected as a model.

#### 2.4.1. Experimental Protein Structure

The crystal structure of FabF protein was retrieved from the RCSB Protein Data Bank (PDB, ID: 6OKG). The structure was downloaded in PDB format and prepared using standard protein preparation protocols (removal of crystallographic water molecules, addition of missing hydrogens, correction of bond orders, and assignment of appropriate protonation states at physiological pH). The structure contained 413 amino acid residues with a biologically relevant conformation suitable for downstream docking studies.

#### 2.4.2. Prediction of the Protein Structure with AlphaFold

To assess the prediction of the protein structure using all protein sequences, the AlphaFold Server [33] was used [25]. The AlphaFold structure was evaluated using: (i) predicted local distance difference test (pLDDT), a per-residue measure of local confidence, estimating how well the prediction would agree with an experimental structure -higher scores indicate higher confidence and usually a more accurate prediction (range: 0 to 100); (ii) Predicted Aligned Error (PAE), a measure of global confidence in AlphaFold predictions, evaluating if the domains are well packed and if the relative placement of the domains in the predicted structure is correct. In the PAE plot, a dark green tile or block corresponded to a good prediction (low error), whereas a light green tile indicated a poor prediction (high error).

#### 2.4.3. Binding Site Prediction

The active-site pocket of FabF was identified based on the location of the catalytic residues and previously reported ligand-bound structures of ketoacyl synthases. Binding-site residues were also cross-validated using cavity detection tools (CASTp/PrankWeb), which consistently predicted a well-defined hydrophobic pocket surrounding the catalytic Cys163. The predicted pocket was lined by residues that contribute to substrate stabilization and inhibitor recognition. The final set of pocket-forming residues was: ILE108, ALA162, CYS163, PHE202, PHE229, MET269, HIS303, THR305, THR307, HIS340, PHE398, GLY399, and PHE400 (Chain A).

#### 2.4.4. Molecular Docking

The ligand cerulenin (PubChem CID: 5282054), a known covalent inhibitor of FabF, was retrieved in SDF format and converted into the docking-compatible format after energy minimization. Since cerulenin forms a covalent adduct with the catalytic cysteine, a non-covalent docking protocol was applied to first estimate the pre-reaction binding orientation. Docking was performed using AutoDock Vina v1.2.6 [33] with a grid box centered around the predicted active-site pocket encompassing CYS163. The scoring function used was calibrated for affinity prediction, and the best-ranked binding pose was selected based on docking score and interaction geometry.

#### 2.4.5. Molecular Interaction Analysis

Protein–ligand complexes were visualized and analyzed using PyMOL 2.6 and LigPlot+ v.2.3 to identify hydrogen bonds, hydrophobic contacts, and proximity-based interactions. Attention was given to interactions involving CYS163, PHE residues, and HIS303/HIS340, which are known to play key roles in substrate and inhibitor binding in FabF. The orientation of cerulenin within the pocket was evaluated to ensure meaningful alignment with the catalytic cavity prior to covalent bond formation.

## 3. Results

**Figure 1:**
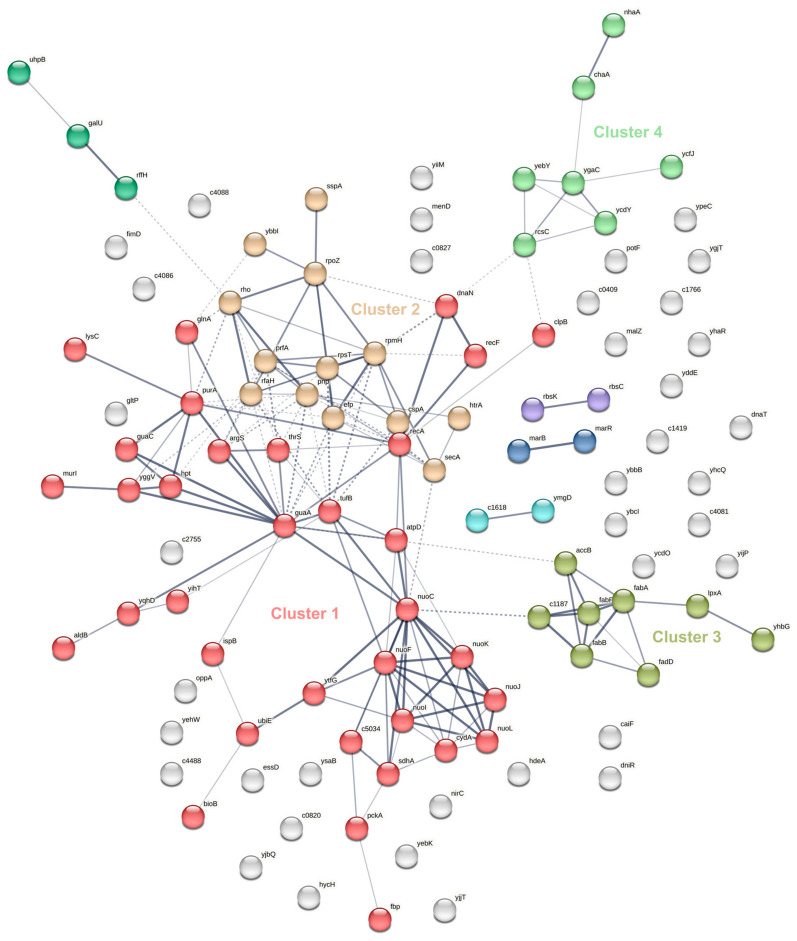
Interactome of all 147 genes belonging to the core perturbomes of *E coli*, *P aeruginosa* and *S. aureus*. Of the 147 genes, 109 were mapped to the interactome database, generating a molecular network with 109 nodes and 169 interactions, significantly higher than the expected number of interactions (PPI enrichment *p*-value = 2.67 × 10^−12^). Clustering analysis (k-means clustering) within the network identified three major functional modules: Cluster 1 (red), associated with oxidative phosphorylation; Cluster 2 (light brown), related to translation, RNA binding, and ribonucleoproteins; and Cluster 3 (green), linked to fatty acid biosynthesis. Additional genes were associated with biosynthetic, transcriptional, and regulatory processes.

**Figure 2:**
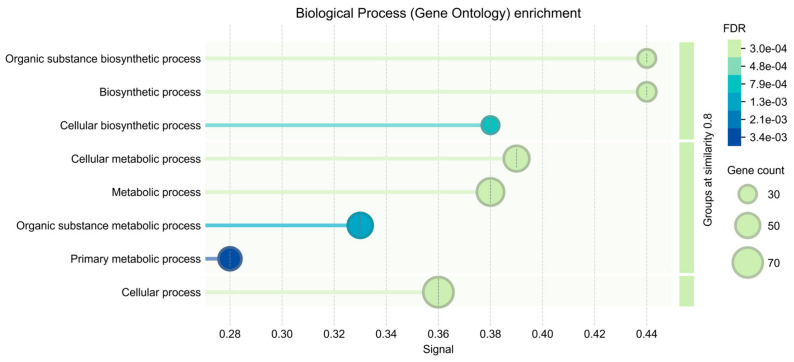
Enrichment analysis for all 147 genes belonging to the core perturbomes of *E coli*, *P aeruginosa* and *S. aureus*. For the genes, different biological functions associated with oxidative phosphorylation, translation, RNA-binding functions, and molecule biosynthesis were highlighted among the enriched metabolic and signaling pathways identified for the studied gene set, including cellular and energy metabolism, biosynthesis activities, and other regulatory processes. Representative genes are indicated by the size of the circles, while statistical significance is represented by the corresponding FDR values.

Core perturbome genes from three bacterial models were retrieved, totaling 147 genes (Table 1). To obtain an overview of the functions associated with these genes and their functional relationships, an interactome construction and gene ontology analyses were performed based on gene sequences (Figure 1 and Figure 2).

Of the 147 initial genes, 109 were successfully mapped to the interactome database, enabling the construction of a molecular network composed of 109 nodes and 169 interactions (Figure 1). This number of interactions is higher than the expected value (94), indicating that the network was not formed by chance, as supported by a highly significant *p*-value (PPI enrichment *p*-value = 2.67 × 10^−12^). On average, each node displayed 3.1 interactions.

Clustering analysis within the molecular network identified several functional modules of interest, highlighting three main clusters that can be associated with metabolic pathways and stress adaptation mechanisms across the three bacterial species analyzed. Cluster 1 (red) included 34 genes primarily associated with oxidative phosphorylation. Cluster 2 (light brown) comprised 13 genes related to mixed functions, including ribonucleoproteins, translation factor activity, and RNA binding. Cluster 3 (green) consisted of 8 genes associated with fatty acid biosynthesis. Other genes in the network were related to additional biosynthetic activities, transcriptional processes, and general regulation. Furthermore, functional enrichment analysis highlighted various biosynthetic and cellular processes at the molecular level, associated with both energy metabolism and other biosynthetic metabolic pathways.

These results are in line with the KEGG functional profile (Figure 2) of this gene set indicates that its main role is centered on core cellular metabolism, particularly fatty acid biosynthesis and energy metabolism, cofactor production, and nucleotide sugar biosynthesis. Overall, the dataset is predominantly associated with central biosynthetic and energy-related processes.

Regarding the identification of orthologs and orthogroups, the sequences from each perturbome were compared. The main functions associated with orthogroups (OG) are reported in Table 1, while Table 2 reports drug–target interactions that were predicted for these elements based on their associated genes. OG0 is related to enzymes involved in the initiation of type II fatty acid biosynthesis, and showed predicted interactions with Cerulenin, Lauric acid, Platensimycin, Capric acid, Caprylic acid, and other chemical compounds. OG1 corresponds to post-translational regulators of amino acid homeostasis and metabolic balance, and interactions were predicted with S-Phosphocysteine and Triglyme. The transcription termination factor Rho, associated with OG2, is reported to interact with Aurovertin B, Piceatannol, Quercetin, Artenimol, and others.

OG5 is involved in glycolipid and cell wall polysaccharide biosynthesis. Interactions were identified with nucleotides and deoxynucleotides, nucleotide sugars (activated sugars), and central metabolic intermediates. In contrast, OG3 (ATP synthase subunit beta) and OG4 (peptidoglycan hydrolase) did not show reported drug–target interaction.

Following the identification of candidate target proteins, structural analyses were performed to evaluate potential drug–protein interactions. Based on the orthogroup analysis, the *E. coli* FabF protein (P0AAI5) was selected as a representative model for in silico characterization.

**Figure 3:**
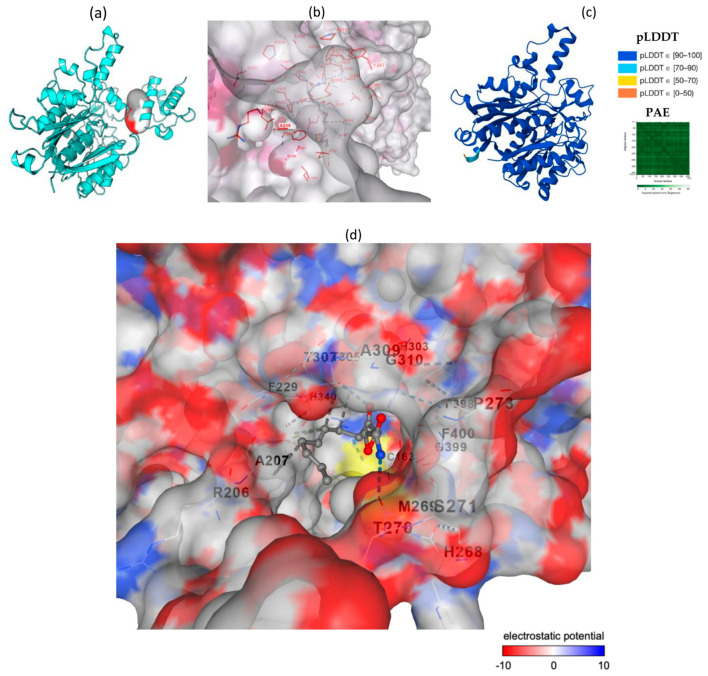
Molecular Docking of FabF and Cerulenin. (**a**,**b**) Three-dimensional structure of FabF (PDB ID: 6OKG) and the predicted active-site cavity of FabF showing the pocket surrounding catalytic residue CYS163 and key pocket-forming residues. (**c**) The AlphaFold model of the FabF based on protein sequence. (**d**) Residue level molecular interaction profile showing contacts between cerulenin and FabF binding-site residues (electrostatic potential is shown).

The three-dimensional structure of FabF (PDB ID: 6OKG) displayed the characteristic β-ketoacyl synthase fold with a clearly defined catalytic cavity (Figure 3a,b). Structural preparation included protonation state assignment and optimization of side-chain conformations to ensure accurate modeling of ligand interactions.

The structure of this protein was also inferred using AlphaFold and high confidence was obtained based on local and global metrics (Figure 3c), in line with the fact that this structure is part of the AlphaFold ground truth dataset, thereby reproducing a high-quality experimentally validated structural model. The average pLDDT was 98, indicating a very high local confidence per-residue and accurate prediction (dark blue), while PAE defined dark-green blocks, suggesting a high global confidence (low error) and well-established domains in the prediction.

**Figure 4:**
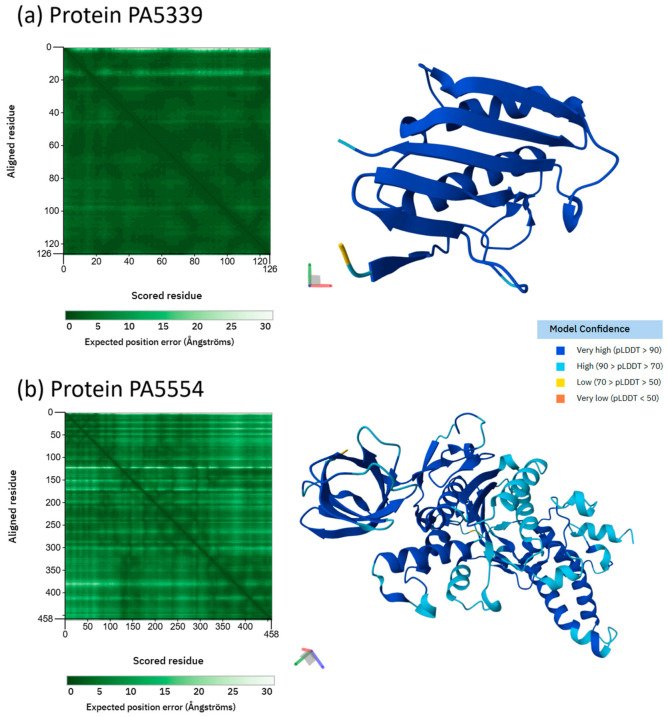
AlphaFold structural prediction of proteins PA5339 and PA5554 lacking experimentally resolved structures in the PDB. Predicted models exhibited high to very high confidence based on average pLDDT and PAE values, supporting the reliability of the global domain organization and overall structural resolution despite the absence of experimental structural data.

This high confidence in AlphaFold structural predictions was consistently observed across all proteins belonging to the identified orthogroups. Notably, although experimental three-dimensional structures are available for most of these proteins, two cases were identified in which no experimentally resolved structures are currently deposited in the PDB database. Interestingly, AlphaFold predictions for these proteins still achieved high to very high confidence levels, as illustrated in Figure 4. For instance, protein PA5339 showed an average pLDDT value of 97.44, while PA5554 presented an average pLDDT value of 90.31, both classified as very high confidence. Although the latter exhibited localized regions with a slight reduction in prediction quality toward the high-confidence category, the overall structural reliability remained robust. These observations were further supported by PAE values, which indicated strong confidence in the global domain organization and structural resolution of both proteins.

Binding site analysis identified a deep hydrophobic pocket surrounding the catalytic residue CYS163, consistent with the known enzymatic mechanism of FabF. The predicted active-site residues included ILE108, ALA162, CYS163, PHE202, PHE229, MET269, HIS303, THR305, THR307, HIS340, PHE398, GLY399, and PHE400. These residues form a continuous cavity capable of accommodating fatty acyl intermediates and known inhibitors such as cerulenin. Pocket architecture combines hydrophobic and polar elements, supporting substrate recognition and catalytic function.

Molecular docking showed that cerulenin binds stably within the FabF active site, with a relative docking score of −5.698 kcal/mol, indicating favorable binding affinity in the pre-covalent conformation. Although this value is relative to the specific docking protocol and scoring function used in this analysis, the more negative binding energies generally indicate stronger predicted interactions and greater complex stability, supporting the idea that cerulenin is able to adopt a stable orientation within the catalytic pocket before covalent bond formation occurs, which is consistent with its known inhibitory mechanism against FabF.

The ligand was oriented such that its reactive epoxide moiety faced the catalytic CYS163, consistent with the experimentally described mechanism in which cerulenin forms a covalent adduct with FabF. The predicted binding pose was also consistent with previously reported FabF–cerulenin complexes (Figure 3a,b).

Interaction analysis revealed that cerulenin engages multiple residues within the binding pocket, primarily through hydrophobic interactions and van der Waals contacts (Figure 3d). Key interacting residues included ILE108, ALA162, CYS163, PHE202, PHE229, MET269, HIS303, THR305, THR307, HIS340, PHE398, GLY399, and PHE400. CYS163 was positioned in close proximity to the epoxide group, supporting its role in covalent inhibition. Aromatic residues (PHE202, PHE229, PHE398, and PHE400) contributed to π–hydrophobic stabilization of the ligand scaffold. HIS303 and HIS340 contributed to the polarity of the pocket and may facilitate ligand orientation, while THR305 and THR307 provided additional stabilizing contacts.

Overall, the docking and interaction analyses support that cerulenin binds in a catalytically relevant orientation within the FabF active site, demonstrating appropriate pre-covalent positioning and consistent engagement with functionally important residues.

## 4. Discussion

Understanding the perturbome as a central stress-response mechanism is key not only to explaining organism survival under adverse conditions but also to identifying potential therapeutic targets applicable across different contexts, with clinical relevance for combating infectious agents [12,34,35]. Recent advances highlight the growing importance of analyzing large-scale perturbations across organisms and making possible the identification of conserved and condition-specific patterns, and guiding target prioritization [10,12]. However, continued dataset curation and expansion remain necessary in the study of response to perturbations [11,12]. Here, we implemented an integrative framework that combines functional analysis, orthogroup identification, structural modeling, and molecular docking to prioritize evolutionarily conserved and biologically relevant targets associated with bacterial stress adaptation, thereby supporting the rational identification of perturbome-informed robust antimicrobial candidates in bacterial organisms.

Based on individual perturbome studies in *E. coli*, *P. aeruginosa*, and *S. aureus*, a total of 147 genes were compiled for integrated characterization to obtain a global view of their functions and relationships. Interactome and gene ontology analyses revealed several relevant functional modules, notably groups associated with oxidative phosphorylation, translation-related functions and RNA binding, and fatty acid biosynthesis. Other genes in the network were linked to additional biosynthetic processes, transcriptional regulation, and general cellular control. All these results are in line with other studies [27,36,37,38,39]. Overall, the functional profile indicates that this gene set is centered on core cellular metabolism, particularly key energy and biosynthetic pathways [7,13]. This supports the idea that the perturbome converges on essential metabolic nodes for bacterial homeostasis and stress response [5,7].

In the second stage, orthologous genes were identified through comparative genomics using the perturbome genes from each species to detect proteins commonly regulated or modulated under different stress conditions. After identifying six conserved orthogroups (with proteins present in at least two species), representative proteins from these groups were used to perform drug–target interaction simulations. The drug–target interactions identified for the orthogroups mainly reflect three types of modulation: direct enzymatic inhibition, interaction with catalytic or regulatory sites, and association with structurally related metabolites that may act as ligands or competitive analogs.

For orthogenes involved in the initiation of type II fatty acid biosynthesis (orthogroup OG0), the drug–target interactions include classical fatty acid synthesis inhibitors such as cerulenin and platensimycin, both described as experimental inhibitors of FAS-II enzymes [40]. Medium-chain fatty acids (lauric, capric, and caprylic acids), some approved or previously used, were also identified and may act as feedback modulators or metabolic competitors. The presence of additional experimental compounds further supports this pathway as a well-recognized antimicrobial target due to its essential role in membrane integrity [41]. In this sense, the initiation of fatty acid biosynthesis and the type II fatty acid elongation cycle are directly involved in lipid biosynthesis and membrane homeostasis [42]. Because the membrane is one of the primary targets of environmental stress, bacteria modulate fatty acid synthesis to preserve appropriate fluidity and permeability. During heat stress, increased synthesis of saturated fatty acids stabilizes the membrane, whereas cold stress promotes unsaturated fatty acid production to maintain fluidity [43]. Under oxidative or antibiotic stress, membrane repair requires active lipid biosynthesis [44]. Thus, fatty acid biosynthesis is both a structural and stress-adaptive biosynthetic process.

In orthogroup OG1, associated with a post-translational regulator of L-threonine metabolism, the predicted interactions correspond to experimental compounds that likely function as metabolic analogs or modulators of phosphorylation or redox balance, potentially interfering with metabolic flux toward toxic intermediates. The post-translational regulator controlling the metabolic fate of L-threonine or the potentially toxic intermediate 2-ketobutyrate is central to metabolic stress control [45,46]. Imbalances in amino acid biosynthesis, nutrient limitation, or redox disturbances can lead to the accumulation of toxic intermediates [44]. Tight regulation prevents metabolic overflow, redirects carbon flux, and minimizes toxicity [47]. This represents a classic metabolic stress buffering system that preserves cellular homeostasis under fluctuating environmental conditions.

On the other hand, the transcription termination factor Rho contributes to transcriptional regulation during stress and indirectly supports biosynthetic balance [48]. Rho prevents aberrant transcription, reduces wasteful RNA synthesis, and maintains genome stability, thereby conserving energy and ensuring that biosynthetic pathways are properly regulated according to environmental demands [49]. Corresponding to orthogroup OG2, the factor Rho was predicted as modulated by a diverse set of experimental, investigational, and some approved inhibitory molecules. This suggests that Rho represents an attractive regulatory node, as its inhibition can globally affect transcription and, consequently, multiple metabolic and stress-response pathways, as supported previously [48].

Unlike other orthogroups, OG3 (ATP synthase subunit beta) and OG4 (peptidoglycan hydrolase involved in septum splitting) show reported drug–target interaction in this dataset, which may reflect limited annotation or lower representation in the interaction database used. However, their roles in the stress response have been studied. ATP synthase subunit beta is directly linked to energy metabolism, which underpins all biosynthetic and stress-response processes [7]. During nutrient deprivation, oxidative stress, or antibiotic exposure, energy requirements shift significantly. ATP is required for macromolecular biosynthesis, protein folding, membrane repair, detoxification systems, and stress signaling pathways [50]. Efficient energy production is therefore fundamental to surviving metabolic stress and sustaining adaptive responses [47,51]. The peptidoglycan hydrolase involved in septum splitting during cell division connects stress to cell wall biosynthesis and remodeling [52]. Under stress conditions, bacteria often slow growth and adjust cell wall synthesis [53]. Because peptidoglycan integrity is critical for survival, especially under envelope or antibiotic stress, hydrolase activity must be tightly coordinated with biosynthesis [52]. Imbalance between degradation and biosynthesis during stress can lead to cell lysis, making this function highly stress-sensitive [54].

The enzyme catalyzing the formation of UDP-glucose from glucose-1-phosphate and UTP links central carbon metabolism to glycolipid biosynthesis and envelope assembly (OG5). Drug–target interactions in this case include nucleotides and activated sugar derivatives (e.g., UDP-glucose, glucose-1-phosphate, thymidine-related compounds), indicating interactions at the active site level or competition with natural substrates, as well as broader metabolic modulation [55]. UDP-glucose serves as a precursor for lipopolysaccharides, teichoic acids, capsules, and exopolysaccharides [56]. During environmental stress, bacteria frequently remodel their envelope and may increase capsule or biofilm production as protective strategies [55]. This highlights the close relationship between carbon metabolism, biosynthesis, and stress adaptation [54,57].

Regarding structural analysis and molecular docking, orthogroup OG0 (fatty acid biosynthesis) was selected as a model, specifically using the experimental protein structure of FabF (P0AAI5) and ligands based on drug–target interactions. The three-dimensional analysis of the target protein was particularly informative, as the structure predicted by AlphaFold showed high concordance with the experimental model, indicating strong confidence according to PAE and pLDDT metrics [25]. This high confidence was also obtained for proteins lacking experimentally resolved structures in the PDB (PA5339 and PA5554), indicating reliable local resolution and global domain organization. This finding is significant given the substantial gap in experimentally resolved structures for many bacterial proteins, even in well-studied organisms [23]. Such agreement highlights an important opportunity to extend perturbome and molecular interaction analyses to other bacterial systems, especially for hypothetical proteins which may account for up to ~40% of bacterial genomes and whose functions remain unknown [21,22]. Although the experimental structure was used for the interaction analyses in this study, cross-validation with predicted models supports the use of structure prediction-based approaches to identify new drug–protein interactions and to guide experimental validation of antimicrobial candidates in future work [20,35].

The docking and interaction analyses support that cerulenin binds in a catalytically relevant orientation within the FabF active site, demonstrating appropriate pre-covalent positioning and consistent engagement with functionally important residues [41]. Cerulenin is a classical covalent inhibitor of ketoacyl-ACP synthases and has long served as a benchmark compound in fatty acid biosynthesis research [58]. Its mechanism involves covalent modification of the catalytic cysteine residue in FabF [40,41]. Similarly, other ligands identified in the drug–target analysis have also been reported as FabF inhibitors, including thiolactomycin (non-covalent inhibitor of the FabB/FabF/FabH enzyme family) and platensimycin (selective FabF inhibitor) [58].

Jointly, this study identified conserved molecular determinants among highly diverse bacteria through comparative analyses, and to our knowledge, this is the first work integrating core perturbomes from different microorganisms to recognize determinants of the stress response and to prioritize potential targets. Despite the biological diversity of the bacterial models and the high experimental variability of transcriptomic datasets generated across different laboratories when core perturbome were previously determined [5,6], it was possible to identify common genes and metabolic pathways associated with bacterial stress responses, reinforcing the biological robustness of these relationships. Thus, findings suggest the existence of conserved mechanisms with a central role in bacterial adaptation, as well as potential shared therapeutic targets.

For future work, experimental validation of specific components remains a priority in order to evaluate not only their potential against the three pathogens analyzed but also their applicability to additional organisms whose perturbomes remain to be characterized. For example, in *P. aeruginosa*, we have conducted experimental analysis using transcriptomic approaches (RNA-Seq) to study the molecular response to antibiotic exposure, identifying genes specifically associated with these perturbations after excluding those belonging to the core perturbome response [27,39]. This approach revealed a potential role for resident phages within the *P. aeruginosa* genome, which was further supported through phenotypic assays as previously reported. Similar strategies are expected to support future experimental validation for the current work in different bacterial models and to evaluate identified targets as potential modulators of bacterial viability.

Among the limitations of this study is the inherent variability in perturbome data generation, which integrates experimental results from multiple laboratories. While this diversity contributes to robustness, it also introduces heterogeneity. Additionally, no organism-specific experimental validations were performed, and the findings are fully dependent on data availability in public repositories and on the computational models employed.

Together, this study based on core perturbomes indicates that bacterial stress responses are integrated physiological adjustments involving coordinated control of biosynthesis, metabolic stress management, transcriptional regulation, energy production, cell division, and envelope remodeling, as previously reported [13,14,15,16]. The predicted drug–target interactions concentrate on essential metabolic enzymes and key transcriptional regulators, reinforcing the idea that core perturbomes converge on strategic biochemical nodes with potential as antimicrobial targets [4,20]. The three perturbomes were previously defined using machine learning approaches, based on transcriptomic data after exposure to several stressors, including antibiotics, phenolic compounds and phytochemicals, bioactive fatty acids, antimicrobial peptides, disinfectants, oxidizing agents, toxic metals, and metabolic inhibitors [5,6].

Thus, this study suggests that several drugs could modulate the perturbome and, consequently, affect the central stress response, potentially compromising bacterial survival. As an integrative approach, conserved and biologically relevant targets associated with bacterial stress adaptation were identified, and it can be considered as a general framework for rational identification of perturbome-informed robust antimicrobial candidates in bacterial systems. Notably, several of the identified compounds have already been recognized for possessing antimicrobial activity, which adds biological coherence to the approach and highlights opportunities for drug repurposing or the development of new therapeutic strategies targeting the core stress-response network.

## 5. Conclusions

This in silico study demonstrates that bacterial stress responses converge on highly conserved metabolic and regulatory elements that are essential for cellular homeostasis and survival. By integrating perturbome datasets from *E. coli*, *P. aeruginosa*, and *S. aureus*, 147 stress-associated genes were functionally characterized, revealing coordinated molecular modules linked to energy production, biosynthesis, transcriptional regulation, and metabolic balance. Importantly, comparative genomics identified six conserved orthogroups representing critical stress-adaptive hubs shared across phylogenetically distinct bacterial pathogens. These included enzymes involved in fatty acid biosynthesis, central carbon metabolism, ATP production, transcription termination (Rho), and cell wall remodeling, highlighting the existence of evolutionarily conserved stress-response mechanisms. Drug–target interaction and molecular docking analyses, particularly for FabF, further supported the biological and therapeutic relevance of these targets, with several associated compounds already recognized for antimicrobial activity. Collectively, these findings provide strong evidence that the bacterial perturbome constitutes an integrated and conserved stress-response network centered on essential biochemical pathways required for adaptation and persistence under adverse conditions. Beyond expanding the understanding of bacterial stress physiology, this work establishes a scalable integrative framework for the identification of conserved perturbome-informed antimicrobial targets with potential broad-spectrum applications. In the context of the growing antimicrobial resistance crisis, these results offer a valuable foundation for the future development of novel therapeutic strategies directed toward highly conserved bacterial vulnerabilities, while also emphasizing the importance of subsequent experimental validation.

## Figures and Tables

**Table 1:** General comparison of the core perturbomes of *E. coli*, *P aeruginosa* and *S. aureus* to identify orthogroups.

Metrics	*E. coli*	*P. aeruginosa*	*S. aureus*
Number of genes in the core perturbome	55	46	46
Number of genes in orthogroups	3	8	3
Number of unassigned genes	52	38	43
Percentage of genes in orthogroups	5.5	17.4	6.5
Percentage of unassigned genes	94.5	82.6	93.5
Number of orthogroups containing species	3	6	3
Number of species-specific orthogroups	0	0	0

**Table 2:** Orthologous genes, functions and possible modulating molecules for the orthogroups obtained in the comparison of core perturbomes of *E coli*, *P aeruginosa* and *S. aureus*.

Orthogroup	*E. coli*	*P. aeruginosa*	*S. aureus*	Function	Possible Modulating Molecules (Drug–Target Interactions)
OG0	P0AAI5	PA1609, PA2965, PA5174	-	Involved in the initiation of the fatty acid biosynthesis, type II fatty acid elongation cycle.	Cerulenin (inhibitor, experimental), Lauric acid (approved), Platensimycin (experimental), Capric acid (experimental), Caprylic acid (approved/withdrawn), Chemical compounds (all experimental): DB08366, DB04302, DB08627, and DB08628.
OG1	P0AGL2	PA5339	-	Post-translational regulator that controls the metabolic fate of L-threonine or the potentially toxic intermediate 2-ketobutyrate	S-Phosphocysteine (experimental),
OG2	P0AG30	PA5554	-	Transcription termination factor Rho	Aurovertin B (experimental), Piceatannol (experimental), Quercetin (investigational), Inositol nicotinate (withdrawn, inhibitor), Phenethyl Isothiocyanate (investigational), Diazoxide (approved, investigational, inhibitor), Artenimol (approved, investigational, ligand), Chemical compound DB08629 (experimental), 1-acetyl-2-carboxypiperidine (experimental).
OG3	-	PA5051	Q5HI60	ATP synthase subunit beta	None
OG4	-	PA1812	Q5HIL2	Peptidoglycan hydrolase involved in the splitting of the septum during cell division.	None
OG5	-	PA5163	Q5HD54	Glycolipid metabolism, catalyzes the formation of UDP-glucose from glucose-1-phosphate and UTP.	Thymidine monophosphate (investigational), Thymidine 5′-triphosphate (experimental), Citric acid (approved, investigational, nutraceutical, vet-approved), Uridine diphosphate glucose (experimental), alpha-D-glucose-1-phosphate (investigational), 2′-Deoxy-thymidine-beta-L-rhamnose (experimental), 2′-deoxy-Thymidine-5′-Diphospho-Alpha-D-Glucose (experimental).

## Data Availability

Processed data supporting the findings of this study are available within the paper and its Supplementary Materials.

## Supplementary Materials

The following supporting information can be downloaded at: https://www.mdpi.com/article/10.3390/pathogens15070665/s1, Supplementary Material S1: Individual perturbomes (protein sequences, fasta format); Supplementary Material S2: Datasets and stressors used to define the core perturbome in three bacterial models (from previous publications) (table format); Supplementary Material S3: Comparison between protein sequences of shared genes within orthogroups (table format).

## Abbreviations

The following abbreviations are used in this manuscript:

pLDDT	Predicted local distance difference test
PAE	Predicted Aligned Error
